# Editorial and Miscellaneous

**Published:** 1865-02

**Authors:** 


### C. S. Medical & Surgical Journal.

RICHMOND, FEBRUARY, 1865.

*E. W. AYRES............PUBLISHER AND PROPRIETOR.*

### EDITORIAL AND MISCELLANEOUS.

*The Medical Profession.*

A late number of the "Saturday Review," contains an article from which the following passages are taken: "Medicine and its professors have long held sway over the hopes and fears of mankind. The science officially taught in universities and lecture-rooms, has over and over again been forced to alter its fundamental principles and its outward practice; yet one-half of mankind has continued to look up with unswerving confidence to the authority of the faculty, while the other half has been ever ready to run after the new sectaries who constantly arise to question the doctrine of the schools, and to propound some new remedy for human suffering. * * * * The plain truth is, that people have followed quacks because they have not found in the doctrines or the practice of the regular profession reasonable ground for confidence. * * * * * If bleeding, calomel, starving, stimulants, warm rooms, open windows, have each been tried in turn, and, as it seems, without any marked advantage one over the other in effecting cures, it was not surprising that skeptics should doubt the inspiration of the oracle whose utterances were found to be so changing. * * * * The doctor, often uncertain of the nature of your disease, was quite ignorant of the cause of it. He had no evidence as to the action of his drug, or even whether it acted at all upon the cause of disease; and lastly, he had no evidence that the drug would effect you in the same manner as others who had taken it. The very utmost that he could urge was a belief, more or less probable, that the same drug had been serviceable in cases presumed to be similar. * * * * * * If science should hereafter gain further insight into the causes of disturbance and the process of restoration, the physician may perchance play a more leading and influential part. As it is, he fills a secondary place, and if he succeeds in averting fresh cause of mischief and in clearing the way for the curative process which is itself beyond his control, he has fully performed his part."

Carlyle, who notwithstanding his singularities, has done more than any living writer to expose "shams," does onor to the truly great, and excites men to do real work, in his remarkable Latter-day Pamphlet on the "Stump Orator," speaks thus of medicine as a profession: "Medicine—guarded too by preliminary impediments and frightful Medusa-heads of quackery, which deter many generous souls from entering, is of the half-articulate professions, and does not much invite the ardent kinds of ambition. The intellect required for medicine might be wholly human, and, indeed, should by all rules be; the profession of Human Healer being radically a sacred one, and connected with the highest priesthoods, or, rather, being itself the outcome and acme of all priesthoods, and divinest conquests of intellects here below. As will appear one day, when men take off their old monastic and ecclesiastic spectacles, and look with eyes again! In essence, the physician's task is always heroic, eminently human; but in practice, most unluckily at present, we find it, too, become in good part beaverish, yielding a money result alone. And what of it is not beaverish,—does not that, too, go mainly to ingenious talking, publishing of yourself; a partly human exercise or waste of intellect, and alas! a partly vulpine ditto? Making the once sacred iatros, or Human Healer, more impossible for us than ever."

Dr. Sibson, F. R. S., President of the Medical Society of London, in his address before the Society, alluding to these passages, remarks: "It is true that our profession does not hold that high and influential position in the eyes of the public that it did formerly. Quackery has at all times been resorted to by many; but it has never been so organized and so much in vogue as now.

"When we find two such able writersand dispassionate men as Mr. Carlyle and the author of the above article in the "Saturday Review," speak of our profession as they do, and that many retired, educated persons, desert the profession and fly to quackery, we are impelled to inquire into the causes of this state of things.

"I shall say a few words first as to the causes which are due to the medical profession itself, and then as to those which may be laid to the charge of the public.

"Amongst causes due to the medical profession itself, may be named the former trust in a great number of medicines, and as a result, their over-use, sometimes to the exclusion of the more important means—diet and regimen; the frequent changes of treatment; the undoubted revolution from a lowering to a sustaining line of practice; the frequent failures of diagnosis evidenced in cases of criminal exposure; the differences of opinion of medical men, often even of those of the most deserved eminence, as expressed in the law courts and before commissioners of lunacy; the occasional habit of one medical man speaking against another to a patient; the practice, unfortunately sanctioned by custom, of the two frequent advertising of medical works in the newspapers; the frequent announcement of new medicines and new plans of treatment, although they have been insufficiently tested; the remarkable general tendency to abandon the old methods of treatment, though based upon practice and observation, and to run after new ones; the unprofessional custom, on the part of one or two medical men, of advertising secret nostrums; the publication of the advertisements of such nostrums in the medical journals, and the common employment of such secret drugs by the profession.

"But while these causes on the part of the profession itself have tended to lower the public estimate of medicine, a far more important series of causes have operated with the public from their own errors. They have habitually expected too much from the medical man. They see judges give erroneous charges and judgments both as to the facts of the case and the law itself; they see the decision of one judge upset by a bench of judges; they see politicians honestly holding the most different opinions with regard to public affairs; they see financiers and economists, both political and social, arriving, after careful study, at opposite conclusions; they find teachers differ in the mode of training the young; and yet, in none of these instances do the public, for a moment, think of reproaching these men—many of them of the highest eminence, the greatest ability, the most perfect integrity.

"Again, in all the sciences, while the great body of opinion is fixed as to the main points, most of the new discoveries and advances are causes of dispute between the most distinguished men of science, and proofs that those eminent men held erroneous views until they were corrected by recent researches; and yet who ever thinks of blaming men of science for the exposure of old errors, or for their differences of opinion? Nay, who does not join in admiration of the unsparing search after truth, which animates this band of fellow-workers? And yet, this freedom from censure which is accorded to the judge when in error, to the statesman, the economist, and the man of science, when they differ from each other in opinion, bring to light new discoveries and root out old errors, is denied by the public to the profession of medicine. And yet how infinitely more difficult is our pursuit than theirs! They are concerned, on the one hand, with more human affairs, patent facts, definite laws, established principles; on the other hand, with single science—limited to one single branch of inquiry, relating generally to inanimate matter, capable of the most penetaating research. But our science embraces all other sciences—it is the knowledge of the highest, the almost inscrutable laws—the divine laws of life itself, with all their marvellous contexture, their complex intricacy, their myriads of parts, and yet their perfect unity. The all weaves itself into the whole; the one works and lives in the other.

"And this applies to the life—the functions of man in health. Let disease seize him, and then how is all that complex harmony rendered still further complex! That difficult machinery deranged; how is the medical man to know with constant precision the seat of the derangement, and the mode of putting it right? And yet if he fail either in the knowledge of disease, or the power of treating it, he is immediately condemned by the public, who are so indulgent to all others. The public, as I said at the opening of the argument, expect from us too much. And why do they do so? Because they are ignorant of the merest rudiments of physiology. But the majority of the public not only distrust the medical profession, who devote themselves with their whole energy to the pursuit of the most difficult of sciences, but they rush into the organized quackery of the day with the most implicit trust. Why is this? It is that they are not only ignorant of physiology, but of the knowledge also of the nature of things, of the great laws of nature, of the necessary chain of cause and effect. They do not feel and know that for any given effect to be produced an adequate cause must be employed. And thus they trust even in the twenty-third dilution of the homœpath—a dilution that, if it were applied to the earth, the sun, and the whole solar system of planets—if these orbs were all broken up and diluted by divisions of a hundred at a time to the twenty-third homœpathic dilution—and if that dilution were placed under the highest power of the microscope, it would be actually invisible!

"Unfortunately this ignorance of physiology and of the knowledge of the nature of things is not limited to the uneducated public; it extends to those who have received a high classical education. It thus applies to some of the highest aristocracy, and to some also of the clergy, who are generally themselves the teachers. If such be the teachers, what must be the knowledge of the taught?

"Another great error on the part of the public is their estimate that our sole function is the giving of drugs; whereas now, as always, though now more than ever, diet, the management of the sick room, the pursuits of life, the mental habits, change of air and scene, exercise, and all that is comprised under regimen and hygiene, are really more effectual and more employed by us than the giving of drugs. Even the "Saturday Review" speaks only of the administering of medicines.

"There are other causes of the popular estimate of our profession due to the errors of the public, but into these I shall not farther inquire, but will attempt to answer very briefly the question: Is our profession worthy of this distrust? If the noble rolls of great dead ones are opened, a long procession of names is brought to view, who, by their high devotion, clear intellect, and searching and laborious pursuit of truth, from the days of Hippocrates to those of Hunter and Laennec, have extended and expanded our knowledg of the laws of life in health and disease, and our power of aiding nature in the restoration of health to those who are suffering from disease. And of those great men, during the last two hundred years, England can claim many of the greatest. Harvey, Sydenham and Hunter enjoy the highest places in all countries.

"But it may be said, these are your great dead, but where are the living illustrious ones? The answer is, that never since the laws of life in health and disease were studied have there been so many earnest inquiries as in our time, whether here, in Germany, France, or even America. Never has the science of medicine, in all its departments, advanced so much as it has done of late years. Our science has every year been becoming more and more of a fixed science. The cross-lights from all other sciences have been brought to our aid, and we can truly say that many of the most important laws of life, both in health and disease, and principles of the treatment of disease, have been searched out, and are now established, so that we have a true standing ground from which future solid advance may take place.

"Animated by the spirit of our great founders, and joining in the earnest pursuit of truth, with a view to the advancement of our truly and eminently human profession, we shall live down calumny, and shall be doing our part towards placing our profession in a higher position than it has ever yet held in the estimation of the public."

---

### *Congress of Geneva.*

Official announcement is now made of the articles agreed upon by the representatives of Belgium, Baden, Denmark, Spain, France, Hesse, Italy, the Netherlands, Portugal, Prussia, Switzerland, and Wurtemburg. The object is one which the members of our profession have often prominently advocated, and which enlightened statesmen have approved. Talleyrand lays down as a principle which should be adopted by civilized nations, the maxim—that nations should do to each o ther in peace the greatest possible amount of good, and in war the least possible amount of harm. The immediate impulse for the Congress was given by M. Henri Dunant, of Geneva, in his book entitled "A Souvenir of Solferino." He had been present as a simple spectator on that 24th day of July, 1859, when more than three hundred thousand men were engaged in combat, when the line-of-battle extended to more than fifteen miles, and the fight lasted more than fifteen hours. He saw, also, during the following days, the sufferings and the privations of the wounded lying on the field or hurried into improvised hospitals, devoured no longer by fire and sword, but hopeless, and dying from being abandoned, from want of ready, sufficient and efficacious help, and from the diseases born of hospitals. He proclaimed anew the conviction that the wounded man on the ground, of whatever nation, is sacred; that humanity is international; and that medical officers in attendance upon the sick and wounded, their assistants, and the stores consecrated to the service of the invalid should be respected. (It will be remembered that when the Confederate army entered Winchester, the Union Hotel Hospital, with its surgeons and assistants (eight) its attendants, nurses, and inmates, all fell into the hands of General Stonewall Jackson, as prisoners of war. So far from destroying the hospital, he directed Brigade-Surgeon Peale to continue in charge, and was informed that all the Federal sick and wounded would be placed under his care. Surgeon Peale accepted the position, and the labors of the hospital were, as before the capture, performed with devotion and skill. The Confederate general went a step further, and presented an example which is not only worthy of record, but also of imitation by the whole civilized world, in warfare. The surgeons and assistant-surgeons were "unconditionally released" upon their "parole of honor," "to report in person, singly or collectively, to the Secretary of War at Washington as such," at the same time pledging themselves to use their "best efforts that the same number of medical officers of the Confederate States army, now prisoners, or who may be hereafter taken, be released on the same terms, and "to have this principle established"—viz: "the unconditional release of all medical officers taken prisoners of war hereafter."—[Ed.]) Encouraged by the favorable reception of his declared convictions, M. Dunant addressed to the War Ministers of nearly all the States of Europe a proposition to send official delegates to Geneva to consider these propositions. Fourteen Governments complied, and, after four days' consultation, their representatives adopted a programme demanding neutralization, during war by belligerant nations, of ambulances and hospitals, of their staff and material; and a common flag and badge for those engaged in the charitable work. M. Dunant, M. Moynier and General Dupin, with others, continued to labor at the practical realization of these objects. Committees were established in the various kingdoms. Commissioners were dispatched to observe the course of events during the war in Schleswig-Holstein, and to ascertain how far voluntary efforts may be made available in mitigating the horrors of war, without interfering with the efficiency of military operations. For a great part of the conception of the authors of this congress is to provide for the organization and official reception of such voluntary charitable corps in time of war. Subsequently they supplicated the Swiss Government, as a neutral Power, to take the initiative in inviting all the sovereign Powers to concert stipulations, which may be introduced into the law of nations, as to the character of the wounded and of those who bring them succor. This invitation has been generally accepted, and has resulted in the important congress from which the present bases of a convention have issued. It is a great work to have sprung so rapidly from the initiative of a few private individuals; and the names of its authors will deserve to be consecrated in the roll of the highest benefactors of our own and future times.

---

DYSPEPSIA.—A Frenchman describes dyspepsia as "The Remorse of a Guilty Stomach."

---

*Surgeon-General of the Federal Army.*

Dr. HAMMOND, the late Surgeon-General of the Federal Army, has been cashiered, and declared incapable of serving again in any public capacity. The report of the Judge Advocate-General sets forth the findings of the Court Martial, the proceedings of which occupied many weeks. They are to the effect that the accused ordered the purveyors to purchase supplies of inferior quality, well knowing them to be such, and to purchase articles at exorbitant prices, with corrupt intent to aid in defrauding the Government, and that he ordered the purchase of "additional large supplies," "corruptly," and "with intent to aid" certain persons "fraudulently to realize large gains thereon." They convict him of official corruption, abuse of power, and a gross breach of public trust. The Court, which was composed of nine general officers, after an investigation of nearly four months, declared him guilty of the charges preferred, and awarded the punishment which, in their judgement, was in accordance with the nature and degree of the offences committed; and a careful examination of the record, says the Judge Advocate-General, leaves no room for doubt as to the validity of the proceedings, or the justness of the findings and sentence.

The following is the order of the President confirming the sentence in this case:

"The record, proceedings, findings and sentence of the Court in the foregoing case are approved; and it is ordered that Brigadier-General William A. Hammond, Surgeon-General of the United States Army, be dismissed the service, and be forever disqualified from holding any office of honor, profit or trust under the Government of the United States.

"A. LINCOLN.

"*August* 18*th*, 1864."

Dr. Hammond has written to the "New York Herald" a letter on the subject, of which the following is a copy:

"WASHINGTON, D. C., Aug. 22, 1864.

"The undersigned has read in the 'Sunday Morning Chronicle' of this city the remarks of Judge Advocate-General Holt on the proceedings of the Court Martial in his case. He learns from this review, and from the order of the President appended, that he has been dismissed the army, and prohibited from ever holding office under the United States.

"The undersigned has no idea that he will lose one friend by this action of the Administration; but his good name is valuable to him, not only as regards those who know him, but those who do not.

"So soon, therefore, as he is furnished with a copy and findings and sentence of the Court, he will present to the public a brief history of the facts leading to his arrest and trial, a review of the record in his case, and some commentaries on the report of the Judge Advocate-General. With these he will be content to submit to the judgement of the world as to how far he has been guilty of the offences charged, and how far he has been the victim of conspiracy, false swearing and a malignant abuse of official power.

"WILLIAM A. HAMMOND."

